# Pathologically phosphorylated tau at S396/404 (PHF-1) is accumulated inside of hippocampal synaptic mitochondria of aged Wild-type mice

**DOI:** 10.1038/s41598-021-83910-w

**Published:** 2021-02-24

**Authors:** Angie K. Torres, Claudia Jara, Margrethe A. Olesen, Cheril Tapia-Rojas

**Affiliations:** Laboratory of Neurobiology of Aging, Centro de Biología Celular y Biomedicina (CEBICEM), Universidad San Sebastián, Carmen Sylva 2444, Providencia, Santiago Chile

**Keywords:** Cell biology, Neuroscience

## Abstract

Brain aging is a natural process characterized by cognitive decline and memory loss. This impairment is related to mitochondrial dysfunction and has recently been linked to the accumulation of abnormal proteins in the hippocampus. Age-related mitochondrial dysfunction could be induced by modified forms of tau. Here, we demonstrated that phosphorylated tau at Ser 396/404 sites, epitope known as PHF-1, is increased in the hippocampus of aged mice at the same time that oxidative damage and mitochondrial dysfunction are observed. Most importantly, we showed that tau PHF-1 is located in hippocampal mitochondria and accumulates in the mitochondria of old mice. Finally, since two mitochondrial populations were found in neurons, we evaluated tau PHF-1 levels in both non-synaptic and synaptic mitochondria. Interestingly, our results revealed that tau PHF-1 accumulates primarily in synaptic mitochondria during aging, and immunogold electron microscopy and Proteinase K protection assays demonstrated that tau PHF-1 is located inside mitochondria. These results demonstrated the presence of phosphorylated tau at PHF-1 commonly related to tauopathy, inside the mitochondria from the hippocampus of healthy aged mice for the first time. Thus, this study strongly suggests that synaptic mitochondria could be damaged by tau PHF-1 accumulation inside this organelle, which in turn could result in synaptic mitochondrial dysfunction, contributing to synaptic failure and memory loss at an advanced age.

## Introduction

Aging is characterized by progressive changes that lead to cellular impairment and functional decline in different tissues, including the brain^1^. Aging is becoming increasingly relevant as life expectancy increases; projecting that by 2050 approximately 22% of the worldwide population will be 60 years or older^2^. In Chile, life expectancy increased 4.2 years per decade in the last 50 years, reaching 80 years old to date. Thus, Chile is one of the countries with the highest increase in life expectancy^3^. Aging is multifactorial and could be influenced by extrinsic environmental factors^4^, complicating our understanding of the mechanisms leading to cell aging. Age is the main risk factor for developing neurodegenerative diseases^5^. Currently, 30% of the aged population suffers from dementia and, unless there are more effective interventions or treatments, prevalence is expected to rise dramatically^5^. In the brain, age-related cell impairment is associated with cognitive decline^6^. The brain uses approximately 20% of the body´s energy^7^ to maintain synapses^8^, and if this energy supply fails it leads to neuronal damage and contributes to the development of pathologies^7^. Mitochondria are the main producer of ATP by oxidative phosphorylation (OXPHOS)^9^ and are fundamental for maintaining redox balance between the production of reactive oxygen species (ROS) and antioxidant defenses^10^. With age, oxidative stress occurs^11^ at the same time that mitochondrial dysfunction is observed^9^. The “free-radicals theory of aging” proposes that aging is a consequence of oxidative damage in cells^12^, but diverse authors have also proposed the mitochondrial hypothesis to explain the aging process^13,14^.

Diverse reports show the accumulation of different proteins in the brain during aging^15,16^, possibly as a consequence of an age-dependent decreased activity of quality control systems^15^, or by post-translational modifications^15^. An example of this is the tau protein, which is widely expressed in the central nervous system (CNS). Under physiological conditions, tau regulates microtubule dynamics depending on its phosphorylation state^17^. However, in pathological conditions tau phosphorylation promotes its aggregation, leading to the development of neurodegenerative disorders^18^. Thus, phosphorylated tau at Ser396/Ser404 sites, epitope known as PHF-1, is a modified form that can induce synaptic failure^19^, the formation of intracellular deposits^20^, and cognitive impairment in different pathologies, including tauopathies and Alzheimer’s disease (AD)^21^.

It is important to highlight that both mitochondrial and synaptic dysfunction are characteristic events of aging^6^, which are reciprocally influenced^22^, and contribute to cognitive impairment^23,24^. Interestingly, in neurons, there are at least two mitochondrial populations, known as non-synaptic and synaptic mitochondria^25^, the latter mitochondrial pool is located in presynaptic terminals, dendritic shafts, or dendritic spines^26^. Synaptic mitochondria are fundamental to synaptic transmission and memory formation^27^, but they are more sensitive to damage than non-synaptic mitochondria^28–31^, and in aged hippocampus fail previous than non-synaptic mitochondria^31–33^. However, it is unknown why this mitochondrial population is more sensitive. Recent studies showed that modified forms of tau induce mitochondrial dysfunction in pathological conditions^34–37^. Most importantly, reports have shown that tau oligomers impair memory by inducing synaptic and mitochondrial dysfunction in wild-type mice^38^; and that overexpression of pseudo-phosphorylated tau potentiates mitochondrial damage induced by neurotoxic agents such as amyloid-beta (Aβ) peptide in mature neurons^39^; but how this occurs is unclear. Interestingly, tau has been detected in mitochondrial extracts from patients and mice models of AD^40^ and it could interact with mitochondrial proteins^41^. However, if phosphorylated tau at PHF-1 sites is located in the mitochondria during aging is unknown; therefore, in this work we evaluated this possibility. We observed oxidative stress and mitochondrial alterations, which occur simultaneously with the increase in phosphorylated tau at the PHF-1 epitope in the hippocampus of aged wild-type (WT) mice. We observed that tau PHF-1 accumulates in a mitochondrial fraction. More specifically, we reported for the first time that tau PHF-1 accumulation occurs mostly in synaptic mitochondria, which was evidenced by biochemical and immunogold assays in hippocampal samples of 18 month-old WT mice. These results strongly suggest that tau PHF-1 is located in the interior of the mitochondria. Finally, and more importantly, using a proteinase K protection assay, we demonstrated that tau PHF-1 is located within the synaptic mitochondria, with a minor proportion in the mitochondrial matrix. Altogether, these results indicate that during normal aging tau is phosphorylated in PHF-1 sites, a modification that promotes its accumulation within synaptic mitochondria. Also, these results strongly suggested that age-related mitochondrial impairment in the synapses could be due, almost in part, to the accumulation of phosphorylated tau PHF-1 in the synaptic mitochondria, possibly inducing age-related synaptic and cognitive impairment. Future studies could address this possibility. This provides an alternative explanation for the cognitive impairment related to memory loss that is widely reported in the aged population.

## Methods

### Animals

3 month-old C57BL/6 mice were obtained from CINBIOT of the Pontificia Universidad Católica de Chile and 18 month-old mice were obtained from Fundación Ciencia & Vida and Institute of Public Health (ISP) from Chile. These animals were housed and maintained at 24 °C on a 12:12 h light–dark cycle, with food and water provided ad libitum. The animals were handled according to the National Institute of Health guidelines (NIH, Baltimore, MD). The animals were anaesthetized using isofluorane in a anesthesia chamber and then euthanized by decapitation. The experimental procedures were approved by the Bioethical and Biosafety Committee of Universidad San Sebastián. This study was carry out in compliance with the ARRIVE guidelines.

### Reagents and antibodies

The primary antibodies used were: mouse anti-β-actin (1:1000, sc-47778, Santa Cruz Biotechnology, Inc.), goat anti-4HNE (1:1000, H6275-02, US Biological Life Sciences), mouse anti-Total OXPHOS Human WB Antibody Cocktail (1:1000, ab110411, Abcam, Inc.), rabbit anti-GAPDH (1:1000, sc-25778, Santa Cruz Biotechnology, Inc.), mouse anti-human tau (1:1000, 2024-10-31, Dako), rabbit Anti-LaminA (1:500, sc-20680, Santa Cruz Biotechnology, Inc.), mouse anti-PSD95 (1:500, sc-32290, Santa Cruz Biotechnology, Inc.), mouse anti-synaptophysin (1:1000, sc-17750, Santa Cruz Biotechnology, Inc.), mouse anti-VDAC (1:1000, sc-390996, Santa Cruz Biotechnology, Inc), mouse anti-tau PHF-1 (phosphorylated at Ser396 and Ser394) was a gift by Dr. Peter Davies (Department of Pathology, Albert Einstein College of Medicine, NY, USA), rabbit anti-TOM70 (1:300, PA5-82508, Thermo Fisher Scientific). The fluorescent dyes used were: MitoTracker Red CM-H2Xros (Catalog number: M7513, Thermo Fisher Scientific), MitoTracker Green FM (Catalog number: M7514, Thermo Fisher Scientific), VECTASHIELD Antifade Mounting Medium with DAPI (Catalog number: H1200, Vector Laboratories, Inc).

### Immunoblotting

Hippocampi of 3 and 18 month-old mice were dissected on ice and immediately processed as previously described (n = 3)^42,43^. Briefly, the hippocampal tissue was homogenized in Triton buffer (5 mM Tris, 150 mM NaCl, 1 mM EDTA, 1% (v/v) Triton X-100, pH = 7.4) supplemented with a protease inhibitor mixture (catalog number 78429, Thermo Fisher Scientific) and phosphatase inhibitors (NaF 20x, Na_2_P_2_O_7_ 300x, Na_3_VO_4_ 100x) using a homogenizer and then sequentially passed through syringes of different calibers. The protein samples were centrifuged at 14,000 rpm for 20 min at 4 °C. The protein concentrations were determined using the BCA Protein Assay Kit (Catalog number 23225, Pierce, Rockford, IL, USA). Samples were resolved by SDS-PAGE, followed by immunoblotting on PVDF membranes. The membranes were incubated with the primary antibodies and anti-mouse, anti-goat, or anti-rabbit IgG peroxidase-conjugated antibodies (Pierce) and visualized using an ECL kit (Luminata Forte Western HRP substrate, Millipore).

### Isolation of a mitochondrial fraction

Hippocampi of 3 and 18 month-old mice were homogenized with MSH buffer (230 mM Mannitol, 70 mM sucrose, 5 mM HEPES, 1 mM EDTA, pH = 7.4) supplemented with phosphatase (NaF 20x, Na_2_P_2_O_7_ 300x, Na_3_VO_4_ 100x) and protease (catalog number 78429, Thermo Fisher Scientific) inhibitors. Lysates were centrifuged at 600 g for 10 min at 4 °C. The pellet was discarded and the supernatant was centrifuged at 8000 g for 10 min at 4 °C. The resulting pellet was resuspended in KCl Respiration buffer (125 mM KCl, 0,1% BSA, 20 mM HEPES, 2 mM MgCl_2_, 2.5 mM KH_2_PO_4_, pH = 7.2) to measure ETC activity or in MSH buffer without EDTA for western blotting.

### Estimation of mitochondrial complex activity

The activity of the mitochondrial complex I and III were estimated by measuring the produced amount of ROS and ATP production in hippocampal mitochondrial enriched preparations. Mitochondrial ROS production was measured using 25 μM of CM-H2DCFDA (DCF) (485 nm, 530 nm) in the Biotek Synergy HT plate reader as previously described^31,43,44^. Isolated mitochondria (25 μg of protein) were added to 100 μl of KCl respiration buffer with 5 mM pyruvate and 2.5 mM malate as oxidative substrates and incubated at 37 °C. ROS production was calculated as the maximum DCF fluorescence following 30 min of incubation, expressed in arbitrary fluorescence units. After ROS measurements, KCl respiration buffer containing mitochondria was centrifuged at 8000 *g* for 10 min at 4 °C and then the ATP concentration was measured in the supernatant using the luciferin/luciferase bioluminescence assay kit (ATP determination kit no. A22066, Molecular Probes, Invitrogen)^31,43–45^.

### Measurement of ROS content

ROS production was measured using the fluorescent dye CM-H2DCFDA (catalog number C6827, Thermo Fisher Scientific). Briefly, hippocampal samples, diluted in Respiration Buffer, were added to a black 96-well plate in duplicate followed by the addition of 25 μM DCF. Then, the plate was incubated for 5 min and read in BioTek Synergy HT (485 nm, 530 nm).

### Measurement of ATP concentration

The ATP concentration was measured in the obtained hippocampal tissue lysates (ATP content in the hippocampus) with Triton buffer (5 mM Tris, 150 mM NaCl, 1 mM EDTA, 1% (v/v) Triton X-100, pH = 7.4). ATP produced by isolated mitochondria incubated with oxidative substrates was evaluated in the supernatant of isolated mitochondrial fractions. In both cases, ATP was measured using a luciferin/luciferase bioluminescence assay kit (ATP determination kit no. A22066, Molecular Probes, Invitrogen)^31,43–45^. The amount of ATP in each sample was calculated from standard curves and normalized to the total protein concentration.

### Hippocampal slices, staining with mitochondrial fluorescent dyes and immunofluorescence

The brains of 3 and 18 month-old mice were dissected and immediately frozen at − 150 °C. The frozen brains were mounted using Optimal cutting temperature compound (OCT compound) in a cryostat at − 22 °C, then coronal 25-µm-thick slices of unfixed hippocampal tissue were obtained. Hippocampal slices were mounted on glass slides and incubated as previously described with mitochondrial fluorescent dyes^43,46,47^. First, the slices were washed three times for 5 min in PBS and then incubated with MitoTracker Green FM to measure mitochondrial mass^43,46,47^ and MitoTracker Red CM-H2Xros to determine mitochondrial membrane potential^43,46,47^. All these dyes were diluted in Krebs–Ringer–Hepes-bicarbonate (KRH) buffer (136 mM NaCl, 20 mM HEPES, 4.7 mM KCl, 1.5 mM MgSO_4_, 1.25 mM CaCl_2_, 5 mM glucose; pH = 7.4) and incubated for 45 min at 37 °C. After incubation, slices were washed three times for 5 min in PBS and mounted with fluorescent mounting media with DAPI (Vector Laboratories Inc, CA, USA). For immunofluorescence, the slices were fixed with Paraformaldehyde (PFA) 4% for 10 min. The primary antibody was incubated overnight (O.N) at 4 °C while the incubation with the secondary antibody was for 1 h at room temperature. Images were acquired on a TCS SP8 laser-scanning confocal microscope (Leica Microsystems, Wetzlar, Germany).

### Synaptic and non-synaptic mitochondria isolation

Hippocampi from 3 and 18 month-old mice were dissected on ice in Isolation Medium (IM: 0,4 M sucrose, 150 mM mannitol, 2 mM EGTA, 10 mM HEPES, pH = 7.4) as previously described^31,48^. Hippocampi were lysed in a glass homogenizer in 7.5 ml of IM buffer. Then, the samples were centrifuged twice at 1300 g for 3 min. The supernatant was then centrifuged at 21,200 g for 10 min. After centrifugation, the pellet was resuspended in Percoll 15% and layered in a Percoll gradient (15–24–40%), which was centrifuged at 30,700 *g* for 8 min. Band 2 (synaptosomes) and band 3 (non-synaptic mitochondria) were separately removed from the density gradient. Each fraction was placed in separate tubes and incubated with digitonin 0.02% for 10 min^48^. Then, both fractions were centrifuged at 16,700 *g* for 10 min. The synaptosomal fraction was placed in a Percoll gradient (15–24–40%) where band 3 (synaptic mitochondria) was extracted and centrifuged at 16,700 *g* for 10 min. Both mitochondrial fractions were resuspended in IM buffer containing 10 mg/ml BSA and centrifuged at 8500 *g* for 10 min. The final pellet was resuspended in Respiration Buffer (125 mM KCl, 0,1% BSA, 20 mM HEPES, 2 mM MgCl_2_, 2.5 mM KH_2_PO_4_, pH = 7.2), and the proteins were quantified and analyzed for PHF-1 by western blot.

### Transmission Electron Microscopy (TEM)

3 and 18 months-old mice were perfused with paraformaldehyde 4% and then the brains were removed (n = 3). Hippocampal coronal sections were obtained and post-fixed with glutaraldehyde 2.5%. Later, the samples were processed and visualized according to the recommendations of the Facility from the Pontificia Universidad Católica de Chile. Briefly, the hippocampal CA1 region was isolated and fixed in 3% glutaraldehyde in 50 mM cacodylate buffer (pH 7.2) for 3 days at room temperature and then post-fixed with 1% osmium tetroxide in cacodylate buffer for 90 min. The slices were then treated with 1% aqueous uranyl acetate, dehydrated in acetone, and embedded in Epon resin. Ultrathin sections were placed on 300-mesh copper electron microscopy grids, stained with uranyl acetate, and examined in a Phillips Tecnai 12 transmission electron microscope (Philips Electron Optics, Holland) at 80 kV. Morphometric analyses of TEM images were performed with the Fiji software. Swollen mitochondria, average area, and intact membrane mitochondria were defined as previously described^31,49^.

### Immunogold electron microscopy (IEM)

3 and 18 month-old mice were perfused with paraformaldehyde 4% and then brains were removed (n = 4). Hippocampal coronal sections were obtained and the CA1 region of the hippocampus was isolated and processed by the Facility of the Pontificia Universidad Católica de Chile. Briefly, samples were fixed in a solution containing 4% paraformaldehyde, 0.5% glutaraldehyde in PBS 0.1 M for 4 h at 4 °C. They were then subjected to a series of dehydration solutions with ethanol 30–50–95–100% and left in a 1:1 solution of ethanol: LR-White overnight. The tissue was embedded in resin for 4 h and then polymerized with gelatin. Ultrathin sections were placed in a nickel grid. The grids were treated with a blocking buffer composed of PBS-BSA 5% for 20 min. They were then washed for 5 min, three times, with PBS-BSA 5% + Triton 0.05% (Wash Buffer) and incubated with the mouse anti-tau PHF-1 primary antibody diluted in PBS-BSA 0.1% (Antibody Buffer) for 1 h at room temperature. After washing three times, the sections were incubated with the secondary antibody conjugated with colloidal gold beads (Goat anti-mouse, EM-grade 15 nm, #25133, Aurion Immunogold Reagents; diluted in antibody buffer) for 1 h at room temperature. Controls for nonspecific binding of the secondary antibody were performed by omitting the primary antibody. Finally, sections were washed in distilled H_2_O and contrasted/embedded in aqueous uranyl 1%. Grids were examined in a Phillips Tecnai 12 transmission electron microscope (Philips Electron Optics, Holland) at 80 kV, from the facility of the Pontificia Universidad Católica de Chile. Finally, images were analyzed in Fiji Software.

### Proteinase K protection assay

Mitochondrial fractions of 18 month-old mice were treated with Proteinase K (0.5 or 0.25 μg/ml) in the presence of buffer, 0.1% digitonin, or 1% Triton X-100 for 15 min at room temperature. We observed the same result with both concentrations. The control condition was performed in the absence of Proteinase K. Later, the protease inhibitor was added to inactivate Proteinase K for 5 min, and then 1 × loading buffer was added before boiling the samples to perform an SDS-PAGE.

### Image analysis

All slides were photographed and scanned under the same magnification, laser intensity, brightness, and gain. Images were processed using the Fiji software (NIH Image)^50^, adjusting the fluorescence threshold intensity in every picture.

### Statistical analysis

The results are presented as bar graphs indicating the mean ± standard deviation. Statistical significance was determined using one-way ANOVA with Bonferroni’s post-test. *p*-values > 0.05 and ≤ 0.05 were regarded, respectively, as not statistically significant and as statistically significant. In the figures, *p*-values between 0.01 and 0.05 are marked with one asterisk, *p*-values between 0.001 and 0.01 with two asterisks, and *p*-values less than 0.001 are shown with three asterisks. All statistical analyses were performed using Prism software (GraphPad Software, Inc.).

### Ethical approval and consent to participate

The experimental procedures were approved by the Bioethical and Biosafety Committee of Universidad San Sebastián.

## Results

### Oxidative balance and mitochondrial bioenergetics function are impaired during aging

**Figure 1:**
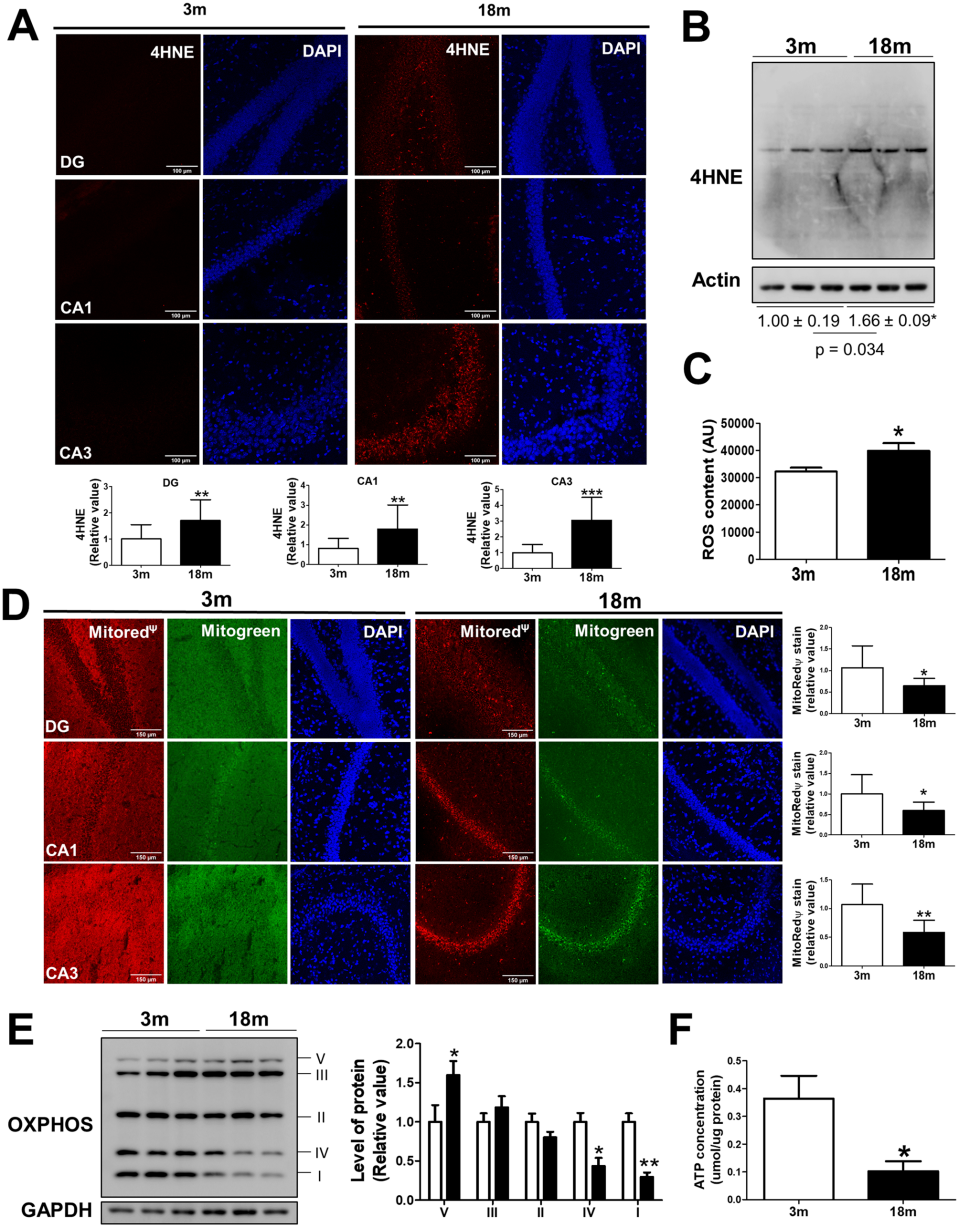
Oxidative damage and mitochondrial dysfunction in the hippocampus during aging. Analysis of oxidative damage indicated by lipoprotein oxidation in the hippocampus of 3 and 18-month-old (mo) mice, using anti-4-hydroxynonenal (4HNE) antibody. (**a**) Representative immunofluorescence images (20x) of three hippocampal regions: Dentate Gyrus (DG), Cornu Ammonis 1 (CA1), and Cornu Ammonis 3 (CA3) and its quantitative analysis. (**b**) Western blot for 4-hydroxynonenal (4HNE) in hippocampal tissue of 3 and 18 mo mice with their densitometric analysis, expressed relative to the control. (**c**) ROS content in the hippocampal tissue, measured by the fluorescent dye CM-H2DCFDA. (**d**) Representative images of non-fixed hippocampal slices stained with Mitotracker Green FM, as a mitochondrial mass indicator, and with MitoTracker Red CM-H2Xros (MitoRedΨ), as a mitochondrial membrane potential indicator. Quantitative analysis of fluorescence intensity was performed in DG, CA1, and CA3 regions of the hippocampus (20x). (**e**) Western blot of oxidative phosphorylation protein complex (OXPHOS) levels. Densitometric analysis is expressed as levels relative to the control. (**f**) ATP content in the hippocampal tissue of 3 and 18 mo mice, measured with an ATP Bioluminescence detection kit. n = 3 different animals of each age. Graph bars represent means ± SEM. *p < 0.05. **p < 0.01; ***p < 0.001. *DG* Dentate Gyrus, *CA1* Cornu ammonis 1, *CA3* Cornu Ammonis 3.

The free radical theory of aging relies on the fact that biomolecules can be damaged by ROS accumulation, leading to functional cell loss^51^. In the brain, mitochondria are one of the main producers of ROS through complexes I and III^11^ therefore, an age-associated mitochondrial dysfunction can lead to increased ROS production and cellular impairment. Here, we evaluated the oxidative damage in the hippocampus of adult mice (3 month-old) and old mice (18 month-old). We assessed the levels of a known oxidative marker 4-Hydroxynonenal (4HNE), which is the product of lipid peroxidation of lipoproteins, by immunofluorescence (Fig. 1a) and western blot (Fig. 1b). In both cases, we observed increased 4HNE levels in the hippocampus of 18 month-old (mo) mice (Fig. 1a,b). Specifically, immunofluorescence assays revealed an increased 4HNE signal in the three studied hippocampal regions: dentate gyrus (DG), cornu ammonis 1 (CA1), and cornu ammonis 3 (CA3) (Fig. 1a). We measured ROS content in the hippocampal lysate, using the fluorescent dye CM-H2DCFDA, and we also observed increased ROS levels in aged mice (Fig. 1c), indicating oxidative stress in the hippocampus during normal aging. We then evaluated the bioenergetic mitochondrial function in the hippocampus of 3 and 18 mo mice. We measured the mitochondrial membrane potential in non-fixed hippocampal slices using the fluorescent dye Mitotracker Red CM-H2Xros, a highly sensitive dye to changes in mitochondrial potential^43,46,47^. We observed decreased fluorescence in DG, CA1, and CA3 hippocampal regions of 18 mo mice (Fig. 1d), indicating depolarization of the mitochondria in the hippocampus of mice at an advanced age. In addition, we evaluated the expression of the oxidative phosphorylation complexes in samples of 3 and 18 mo mice by western blot. Our results reveal significantly decreased levels of complex I and complex IV, accompanied by increased levels of complex V in hippocampal samples of aged mice (Fig. 1e). Finally, we evaluated the ATP content in the hippocampal lysate of 3 and 18 mo mice and observed a significant decrease in ATP in old mice (Fig. 1f). Altogether, these results suggest impaired bioenergetic function of hippocampal mitochondria during aging, which could at least partially explain the oxidative damage present in the aged hippocampus.

### Severe structural alterations in the mitochondria during normal aging

**Figure 2:**
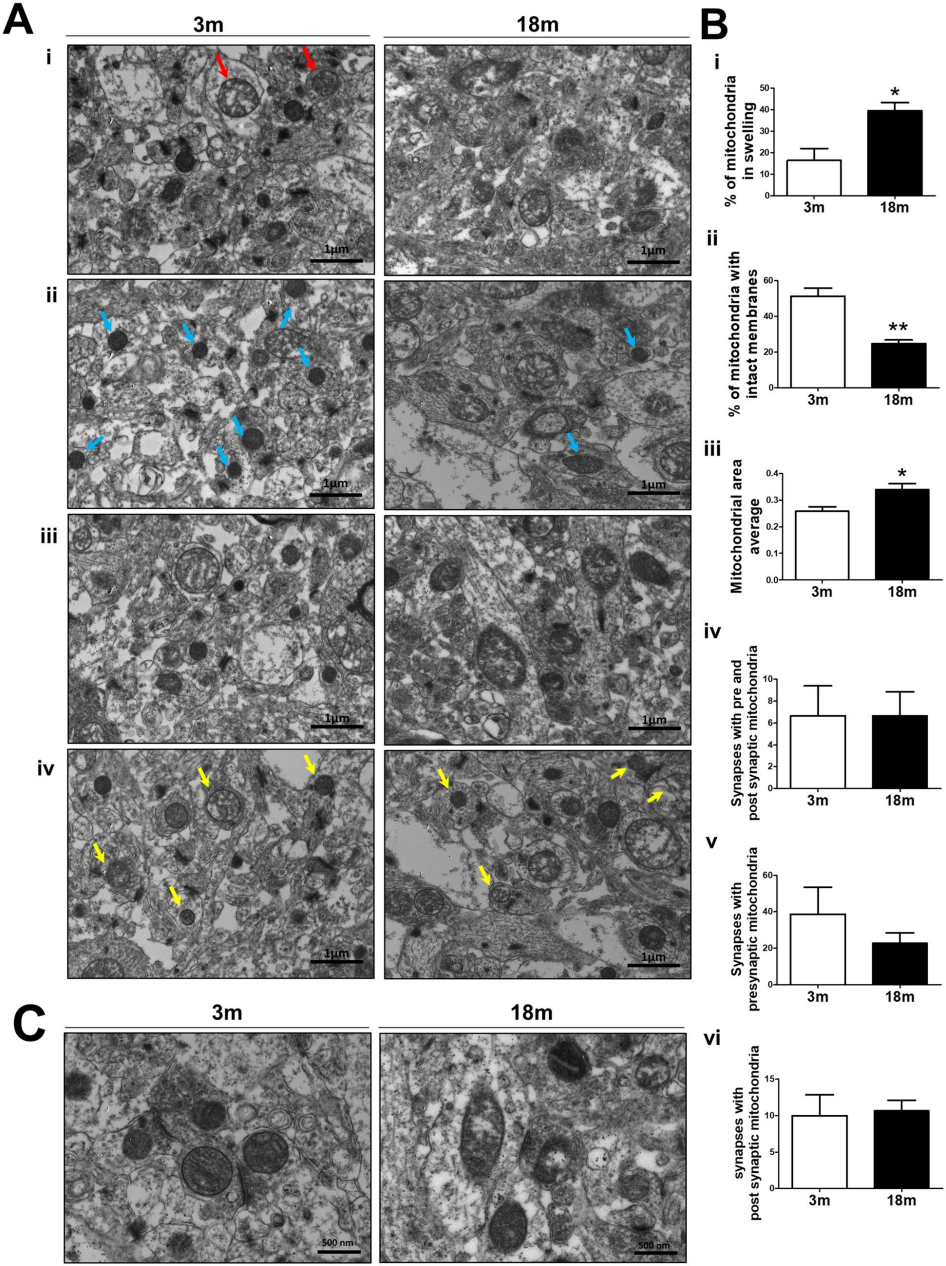
Hippocampal aging increased mitochondrial swelling and reduced mitochondrial integrity. Transmission electron microscopy (TEM) of the hippocampal CA1 region of 3 and 18-month-old (mo) mice. (**a**) Representative TEM images showing (i) mitochondrial swelling, (ii) mitochondrial intact membrane, (iii) average mitochondrial area, and (iv) synaptic mitochondria in both 3 and 18 mo mice (26,500x). Red arrows show swollen mitochondria and light blue arrows show mitochondria with an intact membrane. Yellow arrows show both pre-synaptic and post-synaptic mitochondria. (**b**) Quantitative analysis of (i) mitochondrial swelling, (ii) mitochondrial intact membrane, (iii) mitochondrial average area, (iv) synapses containing pre- and post-synaptic mitochondria, (v) synapses with pre-synaptic mitochondria and (v) synapses with post-synaptic mitochondria was performed. n = 3 different animals of each age. Graph bars represent means ± SEM. *p < 0.05. (**c**) Higher magnification of 3 and 18 month old hippocampal synaptic mitochondria.

Mitochondria are double-membrane organelles organized as an interconnected network^52^. The mitochondrial bioenergetic function is related to mitochondrial morphology^31,53^; and therefore impaired mitochondrial integrity can, in turn, affect their activity; leading to degenerative processes^31,54^. Since old mice have impaired mitochondrial function, we evaluated the mitochondrial structure in the CA1 hippocampus of 3 and 18 mo mice by transmission electron microscopy (TEM) (Fig. 2). We evaluated mitochondrial swelling, mitochondrial intact membranes, average mitochondrial area, and the number of synaptic mitochondria (Fig. 2), using different parameters as previously described^49^. Our results showed an increased percentage of swollen mitochondria (Fig. 2ai,bi) and a decreased percentage of mitochondria with an intact membrane in 18 mo mice compared with 3 mo mice (Fig. 2aii,bii). We also observed increased mitochondrial area in aged mice (Fig. 2aiii,biii). Interestingly, when we evaluated the number of synapses containing both pre-synaptic and post-synaptic mitochondria, we observed no significant difference between 3 and 18 mo mice (Fig. 2aiv,biv): we obtained a similar observation when we compared the number of synapses containing only mitochondria in either the pre-synaptic or post-synaptic region (Fig. 2bv,bvi). Taken together, these results indicate that mitochondria of the CA1 hippocampus from 18mo mice lose their structure with no changes in their distribution throughout the synapses. Therefore, this could be directly related to the negative changes observed in the bioenergetic function.

### Phosphorylated tau at the PHF-1 epitope is increased in the hippocampus of aged mice

**Figure 3:**
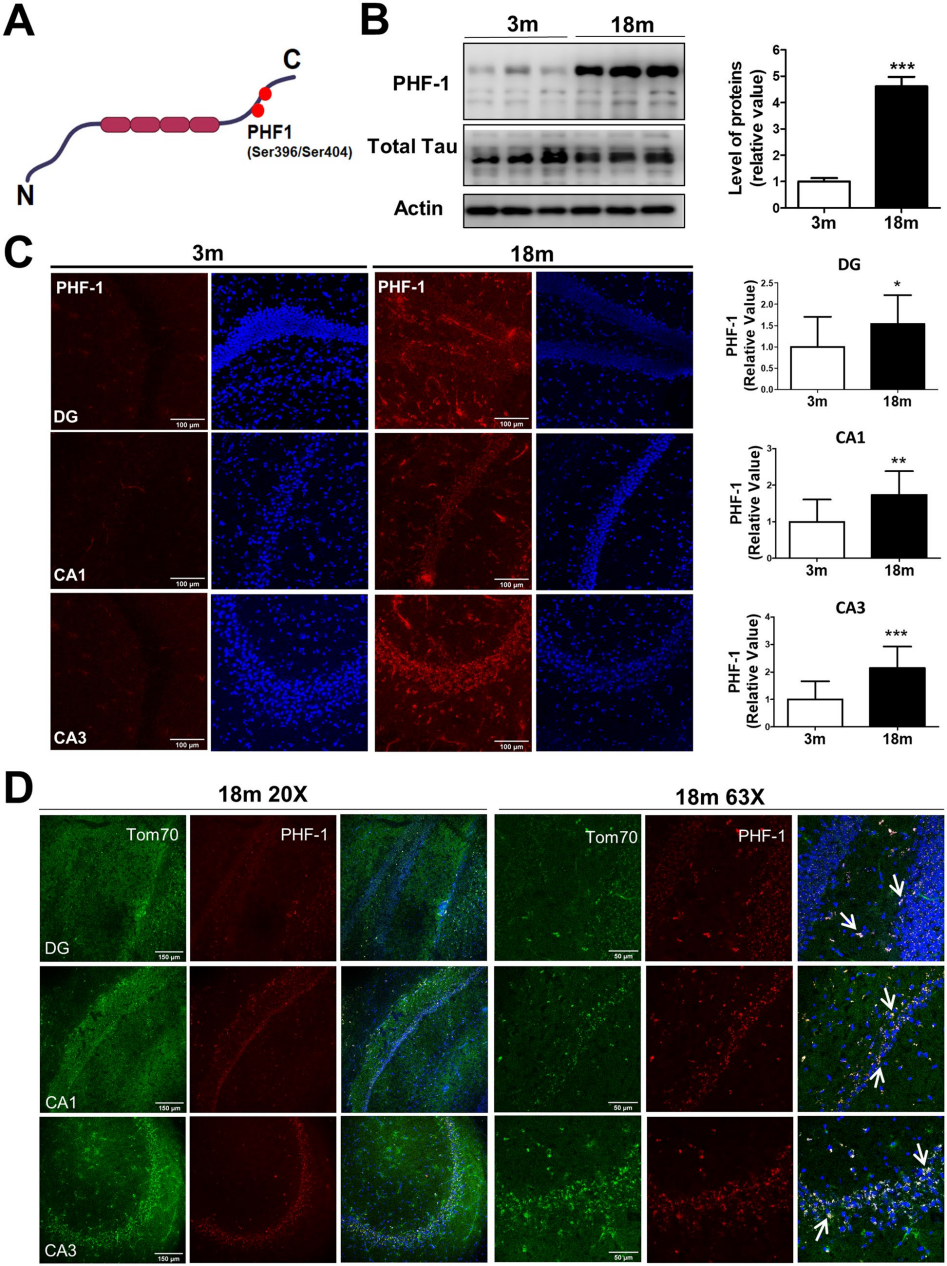
Phosphorylated tau at Ser396/404 (PHF-1) epitopes increased during aging in the hippocampus. (**a**) Schematic representation of tau protein and the phosphorylation at Ser404/396 residues, epitope known as PHF-1. Created with BioRender.com, (**b**) Western blot of tau PHF-1 in hippocampal tissue of 3 and 18-month-old (mo) mice. Densitometric analysis is shown as relative values to the control. (**c**) Representative immunofluorescence images for PHF-1 tau DG, CA1, and CA3 hippocampal regions, with its quantitative fluorescence analysis. (**d**). Double staining for Tom70 (mitochondrial marker; green) and PHF-1 (Red) to analyze the colocalization of phosphorylated tau with mitochondria, in DG, CA1 and CA3 hippocampal regions. White arrows show co-localization between PHF-1 tau and mitochondria.The images were taken using a (**c**) 20 × objective and (**d**) 63 × objective. n = 3 different animals of each age. Graph bars represent means ± SEM. *p < 0.05. **p < 0.01; ***p < 0.001. *DG* Dentate Gyrus, *CA1* Cornu ammonis 1 and *CA3* Cornu Ammonis 3.

Considering that abnormal forms of tau produce mitochondrial dysfunction^55^, we evaluated phosphorylated tau at the PHF-1 epitope (Fig. 3). This phosphorylation is present in different tauopathies^21^. Therefore, we evaluated PHF-1 expression using a specific antibody that simultaneously recognizes both phosphorylated residues (Fig. 3a) in the hippocampus of 3 and 18 mo mice by western blot in a whole lysate (Fig. 3b) and through immunofluorescence (Fig. 3c). Interestingly, we observed a drastic increase in the tau PHF-1 levels, with no changes in total tau levels in old mice (18 m) compared with 3 mo adult mice (Fig. 3b). Consistently, we found increased tau PHF-1 positive signals in the three analyzed hippocampal regions (DG, CA1, and CA3) of aged mice (Fig. 3c), suggesting that this modified form of tau accumulates in the hippocampus during aging. Next, we evaluated the possible localization of tau PHF-1 in the hippocampus. For this, we performed a co-immunofluorescence for Tom70 (mitochondrial marker) and tau PHF-1 in the hippocampal slices. Surprisingly, we observed co-localization of tau PHF-1 with the mitochondria, as indicated by white arrows in 63 × images in Fig. 3d. This last result suggests that tau PHF-1 accumulation during aging could occur in the hippocampal mitochondria.

### Tau phosphorylation at Ser396/404 (PHF-1) is increased in a mitochondrial fraction from the hippocampus of 18 month-old mice

**Figure 4:**
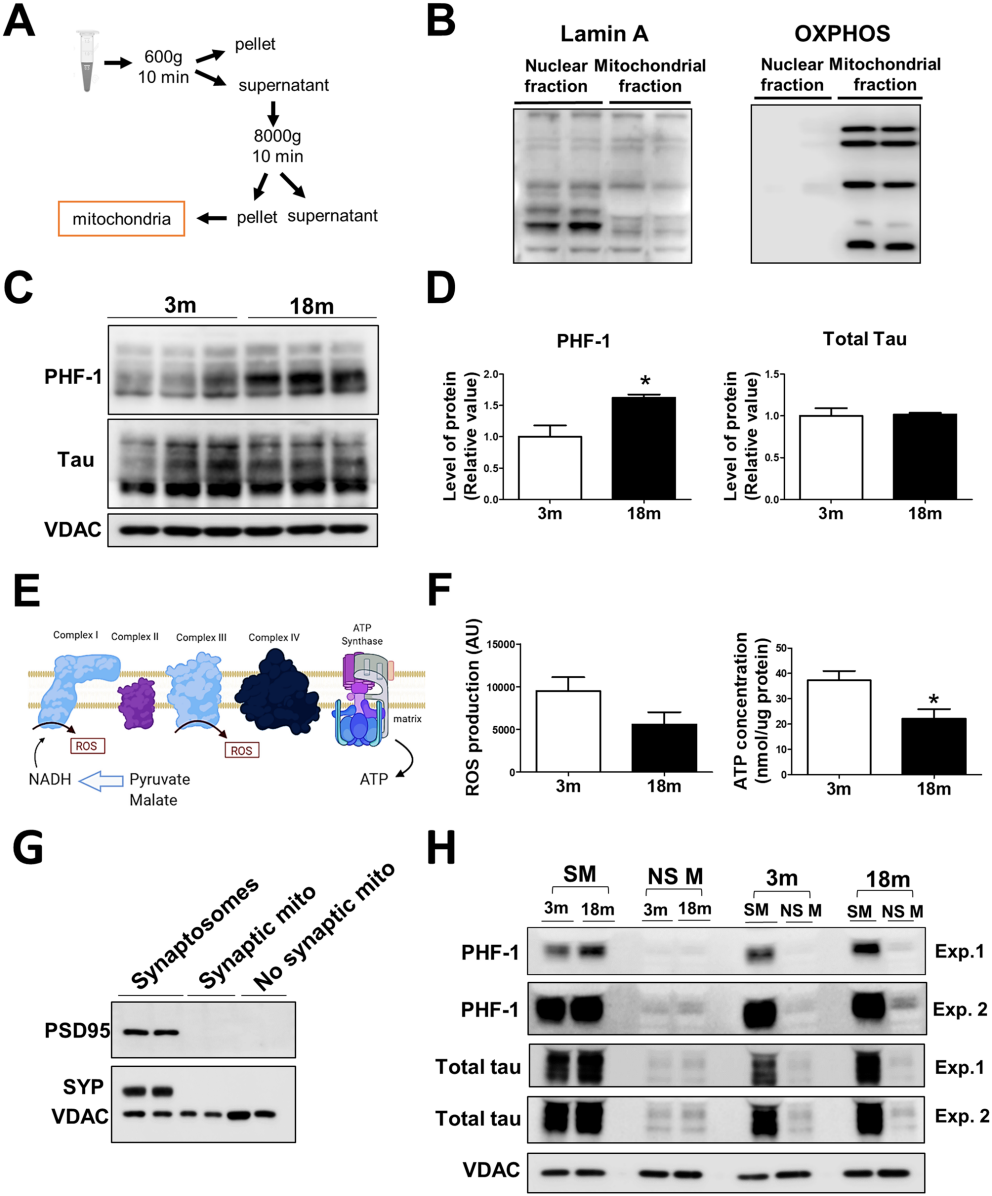
Tau PHF-1 accumulates in hippocampal mitochondria of aged mice. (**a**) Schematic representation of the protocol to obtain a mitochondrial fraction. (**b**) Controls show an enriched mitochondrial fraction. (**c**) Phosphorylated tau at PHF-1 epitope and total tau levels in 3 and 18 month-old (mo) mice were analyzed in a mitochondrial fraction. (**d**) Densitometric analysis of tau PHF-1 and total tau, expressed as a relative value to the control. (**e**) Schematic representation of functional analysis of the mitochondrial fraction using pyruvate and malate as oxidative substrates to stimulate respiration. Created with BioRender.com (**f**). ROS production of isolated mitochondria measured by the fluorescent dye CM-H2DCFDA (to indirectly evaluate the mitochondrial activity of complexes I and III) and ATP production measured by luminescence, both after exposure to oxidative substrates. (**g**) Synaptic and non-synaptic mitochondria were isolated from the hippocampus of 3 and 18 mo mice to measure phosphorylated tau (PHF-1) in both mitochondrial populations. (**h**). Levels of tau PHF-1 and total tau in both synaptic and non-synaptic mitochondria, using VDAC as a loading control. Two different exposure types are showed to demonstrate the reduced levels of both phosphorylated and total tau in the non-synaptic mitochondrial population. n = 3 different animals. Graph bars represent means ± SEM. *p < 0.05.

Different forms of tau induce mitochondrial dysfunction^35^ and in Alzheimer’s Disease tau overexpression has been observed in the mitochondria^56^. However, it is currently unknown if this protein is located in the mitochondria under physiological conditions in vivo during normal aging. To corroborate that tau PHF-1 accumulates in hippocampal mitochondria of old mice, we obtained a fraction enriched in hippocampal mitochondria of 3 and 18 mo mice, using a sucrose buffer and differential centrifugation, as indicated in the scheme of Fig. 4a. To assess if the mitochondrial fraction was correctly isolated, and considering that tau protein has also been described in the nucleus^57^, we performed an immunoblot assay for a nuclear protein (Lamin A) and for mitochondrial resident proteins such as OXPHOS complexes (Fig. 4b), confirming the purity of this mitochondrial fraction. In this mitochondrial fraction, we evaluated the tau PHF-1 and total tau expression (Fig. 4c). We observed that tau PHF-1 levels are significantly increased in the mitochondrial fraction of aged mice compared to 3mo mice (Fig. 4c,d), without significant differences in total tau levels between both ages (Fig. 4c,d). This supports our idea that this phosphorylated form of tau accumulates in the mitochondria during normal aging. Then, we evaluated the bioenergetic function of isolated mitochondria from the hippocampus of 3 and 18 mo mice, measuring ROS and ATP production after the addition of the oxidative substrates pyruvate and malate (Fig. 4e). We observed no significant differences in ROS levels in both age groups; but interestingly our results revealed a significantly reduced ATP production (Fig. 4f), indicating deficient mitochondrial oxidative phosphorylation during aging, which could be mediated, almost in part, by the presence of tau PHF-1 in the mitochondria.

In neurons, two mitochondrial populations with different susceptibility to aging-dependent dysfunction have been described^33^, known as synaptic and non-synaptic mitochondria according to their localization. We evaluated if the accumulation of phosphorylated tau PHF-1 is differential in these two populations. For this, we used a Percoll gradient to isolate non-synaptic mitochondria and synaptic mitochondria from synaptosomes of the hippocampus of adult and old mice (Fig. 4g). Interestingly, when we evaluated the tau PHF-1 expression in both mitochondrial populations, we observed that tau PHF-1 is increased mostly in the synaptic mitochondria of aged mice, with a weak phosphorylated tau signal in the non-synaptic mitochondria, which became more evident with longer exposure time (Exp. 2) (Fig. 4h). Also, we observed that the total tau signal was similar between 3 and 18 month-old mice in both synaptic and non-synaptic mitochondria. Altogether, these results strongly suggest that tau PHF-1 accumulation in the hippocampus of aged mice occurs mainly in synaptic mitochondria.

### Tau PHF-1 accumulates inside synaptic mitochondria of aged wild-type mice

**Figure 5:**
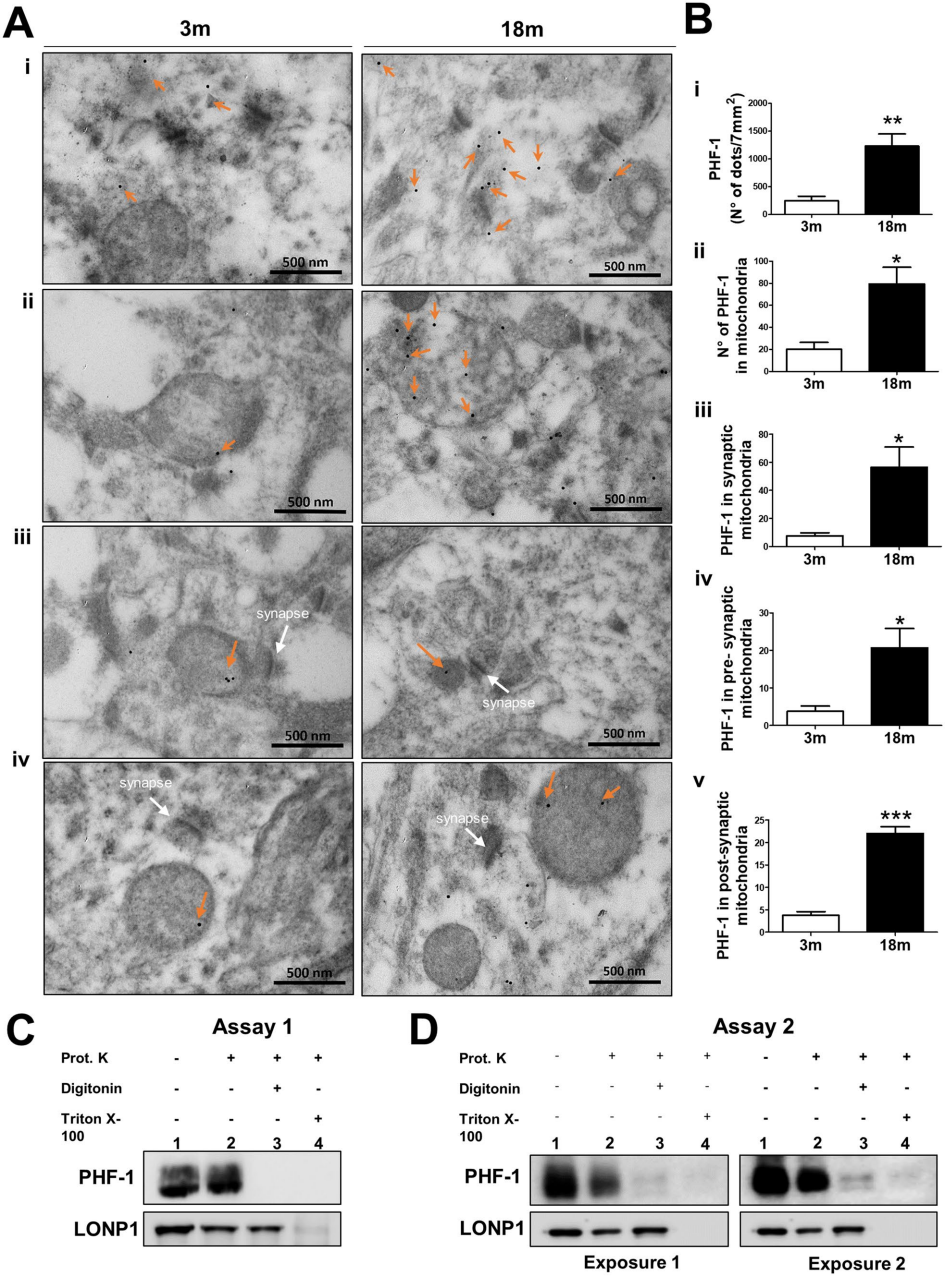
Tau PHF-1 accumulates inside hippocampal synaptic mitochondria at an advanced age. (**a**) Immuno-gold for PHF-1 tau in the hippocampal CA1 region of 3 and 18 month-old mice. Representative images of immuno-gold electron microscopy (IEM) (43,000x) showing (i) the amount of PHF-1 positive signal within an area of the grid of 7 mm^2^; (ii) amount of PHF-1 in the mitochondria, (iii) the number of PHF-1 tau in pre and (iv) post-synaptic mitochondria. (**b**) Quantitative analysis of IEM. (**c**). Western Blot of the proteinase K protection assay in a mitochondrial fraction of 18 mo mice. The mitochondrial fraction was treated with Proteinase K with or without 0.1% Digitonin or 1% Triton X-100. The mitochondrial fraction without Proteinase K was used as a control. (left, Assay 1) Western Blot of the total volume of mitochondrial samples. (Right, Assay 2) Western Blot of 1/3 of the volume of samples 1 and 2. n = 4 different animals. Graph bars represent means ± SEM. *p < 0.05, **p < 0.01.

As mentioned previously, tau PHF-1 appears to accumulate in synaptic hippocampal mitochondria during normal aging. To corroborate these findings, we performed an immunogold electron microscopy (IEM) assay in samples of the CA1 region from the hippocampus of 3 and 18 mo mice (Fig. 5). First, we evaluated the total tau PHF-1 positive signal within one electron microscopy grid (7 mm^2^) (Fig. 5ai,bi). Consistent with our prior results, we observed a drastic increase in the tau PHF-1 signal in old mice (Fig. 5ai,bi). Next, we analyzed the tau PHF-1 positive signal located in the mitochondria (Fig. 5aii,bii). In fact, our results revealed that tau PHF-1 was increased in the mitochondria during aging (Fig. 5aii,bii), supporting our biochemical assays. Finally, we analyzed the amount of tau PHF-1 positive signal in synaptic mitochondria and as expected, aged mice showed higher levels of tau PHF-1 in synaptic mitochondria compared with 3 mo mice (Fig. 5aiii-iv and 5biii-v). Specifically, we observed an increase in the number of both pre-synaptic and post-synaptic mitochondria that were positive for tau PHF-1 in old animals compared with 3 mo animals (Fig. 5biv and 5bv). Interestingly, the tau PHF-1 signal in the mitochondria was observed in the periphery and in the center of this organelle, strongly suggesting that PHF-1 can enter the mitochondria, locating in the intramembranous space or the mitochondrial matrix (Fig. 5aiii–iv). Finally, to validate that tau protein PHF-1 localizes inside the mitochondria during normal aging, we performed a proteinase K protection assay in a mitochondrial fraction of 18 mo mice (Fig. 5c,d). Briefly, we used Digitonin for solubilizing the outer mitochondrial membrane (OMM) and Triton X-100 for solubilizing both the inner (IMM) and outer mitochondrial membrane (OMM). Thus, we analyzed the mitochondrial internal localization of tau PHF-1. In a first assay, we charged the full volume of the obtained mitochondrial fraction and we observed a positive signal for tau PHF-1 only with proteinase K (line 2), an effect that was not observed in presence of Proteinase K plus Digitonin (line 3) or Triton X 100 (line 4) (Fig. 5c). These results suggest that tau PHF-1 is located mainly in the intermembranous space (IMS) of the mitochondria. To corroborate that the absence of signal in line 3 is not because the amount of PHF-1 tau in the mitochondrial matrix is too low, we performed a second experiment where we charged 1/3 of the full volume in the 2 first lines, and the full volume in the last 2 lines (Fig. 5d). Interestingly, and as expected, we observed tau PHF-1 signal in line 3 (digitonin + proteinase K), indicating that PHF-1 is also present in the mitochondrial matrix but in a much smaller proportion. This result is important because it demonstrates that tau PHF-1 is localized inside the hippocampal mitochondria of aged mice.

Taken together, the results of this study showed that phosphorylated tau at the PHF-1 epitope is increased in the hippocampus of mice during normal aging. Even more, we demonstrated for the first time that tau PHF-1 accumulates in the mitochondria of aged wild-type mice, localizing specifically inside of the synaptic mitochondria from the hippocampus at an advanced age.

## Discussion

In the present study, we demonstrate that oxidative stress and mitochondrial alterations occur simultaneously to increased phosphorylated tau at PHF-1 in the hippocampus of aged WT mice. We observed that tau PHF-1 accumulates in a mitochondrial fraction, mostly into synaptic mitochondria, demonstrating that this pathological form of tau PHF1 is located inside of the synaptic mitochondria during normal aging. These results strongly suggested that age-related synaptic impairment could be due to the accumulation of phosphorylated tau PHF-1 in the synaptic mitochondria and propose a mechanism that explains why synaptic mitochondria are more vulnerable to damage during aging^31^.

Aging is the primary risk factor for neurodegenerative diseases that currently lack early diagnosis and cures^58^. This process leads to the decline of cognitive abilities^59^, reducing the quality of life of elderly persons, whose population has increased over the last years. However, the cellular and molecular mechanisms leading to age-associated alterations are not completely clear^60^. Increasing evidence shows that synaptic dysfunction^61^ and mitochondrial impairment^62–64^ are considered early events during aging and in the pathogenesis of neurodegenerative disorders^63^. Also, mitochondrial dysfunction is considered a hallmark of aging^6,63^, because its functional deterioration contributes to the aged phenotype^63,65^. Considering the high-energy requirements of neurons to adequately perform all their functions in the brain, it is logical to hypothesize that an energy imbalance could lead to neurodegenerative pathologies. The main energy producers (ATP) in cells are mitochondria, which maintain the bioenergetic homeostasis of neurons^66^. Also, mitochondria form ROS, which damages neurons when produced in excess^67,68^ leading to the “free-radicals theory of aging” ^69^; but this could be a direct effect of mitochondrial dysfunction. Defects in mitochondrial bioenergetics, as well as disturbances in redox homeostasis, are characteristic signs of neuronal damage in the aged brain. Thus, increased ROS production may trigger a “vicious cycle” of oxidative stress, leading to more severe mitochondrial dysfunction, which contributes to aging^10^. In this context, in this study we showed several impairments in hippocampal mitochondria of aged WT mice, such as increased oxidative stress and bioenergetic impairment, where the increased oxidative stress observed in aged mice could contribute to the impairment of the ETC functioning. We observed a decrease in the expression of the complex I and IV, which are the two complexes that have more subunit encoded in the mtDNA^70^. Thus, the increase in oxidative stress present in the aged hippocampus could induce mtDNA damage, decreasing the expression of these two complexes and leading to the impairment in the ATP production of the aged mitochondria that we also reported. Moreover, we show structural alterations in aged mitochondria of WT mice (increased mitochondrial area and swelling, and decreased membrane integrity), corroborating that aged hippocampus from C57BL/6 mice manifest mitochondrial alterations related to dysfunction in the absence of pathology. The reasons that lead to this dysfunction are still a matter of study, but one possibility is the accumulation of abnormal proteins in the neurons.

Effectively, during aging, accumulations of specific proteins are observed in the brain and can be drastically increased in neurodegenerative disorders^15,71^. Among these accumulations are those formed by tau, a neuronal protein that is involved in tubulin polymerization and microtubule stabilization^72,73^. Tau can be phosphorylated at serine 85 or different threonine sites, many of which are important for regulating its function, and others are associated with pathology^74^. Here, we showed that the levels of a modified form of tau protein are increased in the normal brain. Specifically, we demonstrated that phosphorylated tau PHF-1 accumulates in the hippocampus of aged WT mice. Interestingly, it is reported that oxidative stress contributes to tau phosphorylation in PHF-1 and other epitopes in a mechanism that is still unknown^75,76^. It is reported that oxidative stress can induce tau phosphorylation through the activation of the glycogen synthase kinase-3β (GSK-3β)^77^ and the activation of p38 MAPK^78^. Since PHF-1 is an epitope that is reported to be phosphorylated by GSK-3β^79^, the age-related increased oxidative stress reported here may activate GSK-3β kinase, contributing to higher tau PHF-1 levels in the hippocampus, nevertheless, this hypothesis remains to be determined. In agreement with our results, tau phosphorylation at PHF-1 also has been shown as a characteristic of healthy aging in the brain, where the presence of phosphorylated tau increases with age in animals and non-demented patients, but this change is even greater in AD^80,81^.

When tau is abnormally phosphorylated it dissociates microtubules, losing its normal function and accumulating in neurons^60,82,83^. Tau hyperphosphorylation leads to aberrant self-assembly in insoluble aggregates, accompanied by synaptic dysfunction and neuronal death in a series of neurodegenerative diseases known as tauopathies^19^. In particular, phosphorylation of tau PHF-1 is sufficient to promote microtubule dissociation^84^ and it can accumulate in other intracellular structures, contributing to its dysfunction. Interestingly, this is the first report that demonstrates that phosphorylated tau (PHF-1) can localize into hippocampal mitochondria of aged mice, through biochemical and electron microscopy analyses and the first to propose a possible mechanism to explain, almost in part, the mitochondrial dysfunction observed in the aged hippocampus. Consistent with this observation, diverse studies suggest that the accumulation of modified forms of tau in neurons disrupts mitochondrial function by an unknown mechanism^85,86^. Also, it was recently shown that tau interacts with several mitochondrial membrane-bound proteins including ATP synthase, mitochondrial creatine kinase U-type, and Drp1^41^. However, no study had shown direct evidence that tau, and specifically its phosphorylated form at the PHF-1 site, could be found in the aged mitochondria or pathological conditions. This is a novel and surprising finding that opens the possibility that phosphorylated tau (PHF-1) could be in some way responsible for the mitochondrial dysfunction reported here such as the increase in the redox imbalance and the bioenergetic deficits. This is in agreement with studies that showed that tau PHF-1 induces the Aβ-mediated loss of mitochondrial membrane potential^39^ and that neuronal cultures from KO tau mice prevent this reduction in mitochondrial membrane potential^87^. In addition, tau deletion in WT mice improves mitochondrial function in non-aged mice^43^. Nevertheless, is not clear whether this disruption in mitochondrial function is a direct or indirect effect of tau. Considering that there is a close relationship between mitochondrial structure and function, another possibility is that the accumulation of tau PHF-1 promotes structural alterations reported in hippocampal mitochondria, favoring the presence of disrupted mitochondria. Thus, tau PHF-1 also could induce functional failure of synaptic mitochondria, mainly considering that if IMM and cristae are disrupted the ETC assembly can be affected; which in turn could lead to bioenergetic impairment. It is reported that in AD tau induces defective mitophagy through the decrease of the translocation of Parkin to mitochondria^88^, leading to the accumulation of defective mitochondria. Also, it is reported that phosphorylated tau alters the mitochondrial dynamics in AD, increasing the fission proteins by their interaction with Drp-1, and decreasing the fusion proteins^37^. These results suggest that tau affect mitochondrial morphology in pathological conditions and a similar effect could be occurring in the aging; however, whether tau PHF-1 induces structural changes by any of these mechanisms or it is directly responsible for mitochondrial dysfunction at an advanced age needs to be determined.

Although in neurons tau is mainly located in axons, it is expressed in a lower amount in somatodendritic compartments, including the plasma membrane, nucleus, and significantly lower amounts in dendrites^74^. Tau mainly interacts with cytoskeletal proteins but also communicates either directly or indirectly with other protein types^41^. These include kinases and phosphatases, extracellular proteins, and membrane proteins^41^. Tau directly binds to Fyn kinase and PSD95 in the post-synaptic region, and this interaction is dependent on Tyr18 phosphorylation^74^. Also, tau can interact with synaptophysin, suggesting a role in the pre-synaptic region^41^. Therefore, it is evident that tau plays a role in the synapses, which is in agreement with our findings that tau PHF-1 is located in synaptic mitochondria.

Synapses are sites of high energy demand and calcium variations; therefore synaptic mitochondria are fundamental to maintain bioenergetic homeostasis at synapses, synaptic function, and memory formation^89^. Interestingly, hippocampal synaptic mitochondria are more sensitive to cumulative damage, and their dysfunction occurs previous to non-synaptic mitochondrial failure^31^. Synaptic mitochondria, that are obtained from a synaptosomal extract, come exclusively from neurons^28^; then, alterations in these mitochondria are strictly restricted to neurons and not to another cellular type^10^. Synaptic mitochondria are usually punctuated and isolated, and they are synthesized in the neuronal soma and transported to the axon or dendrite^90^. As a result of this process, these mitochondria are considered older than mitochondria present in the soma of neurons, and they exhibit greater sensitivity to the damage caused by oxidative stress and bioenergetic failure^28^. This sensitivity occurs since synaptic mitochondria possess functional differences compared to non-synaptic mitochondria, such as lower calcium buffer capacity, high ROS production, and lower expression levels of mitochondrial respiratory complexes^91^. Interestingly, aging seems to enhance the differences between these two mitochondrial populations. For example, synaptic mitochondrial extracts of old rats^32^ or mice^33^ presented a significant reduction in mitochondrial respiration, and they were more susceptible to calcium stress^32^ compared to mitochondria of young animals. However, why hippocampal synaptic mitochondria are more vulnerable to cumulative damage is still unknown. Interestingly, our results reveal that phosphorylated tau at PHF-1 is observed mainly in synaptic mitochondria in both non-aged and aged mice, an effect that is drastically increased in aged synaptic mitochondria. Thus, we demonstrated that increased tau PHF-1 levels at an advanced age are located more specifically into synaptic mitochondria and could be involved in the dysfunction of this mitochondrial pool. This is particularly important since the effect of tau PHF-1 accumulation in these mitochondrial population could impact directly in the synapse functioning. Here we show new evidence that is helpful to understand part of the mechanism underlying the previously reported age-related synapse impairment^92^. Tau PHF-1 accumulation inside synaptic mitochondria may play a crucial role in the morphological alterations in these population, which could explain its functional impairment during aging^31^, suggesting that this structural and functional impairment associated with phosphorylated tau accumulation in synaptic mitochondria is a key issue involved in memory loss during aging. More studies are necessary to validate this hypothesis.

## Conclusion

Interestingly, our results reveal that tau PHF-1 is higher located in the periphery of the mitochondria, possibly bound to proteins of the OMM as suggested by interaction assays^41^, in the intermembrane space or bound to proteins of the IMM, with a minor proportion of tau PHF-1 in the mitochondrial matrix. This is particularly relevant because it reveals for the first time that tau PHF-1 can enter synaptic mitochondria during normal aging. Also, this study proposes that tau PHF-1 is located inside the synaptic mitochondria and could trigger the synaptic failure observed in the hippocampus during the early stages of AD and in pathological mouse models and patients.

## Supplementary Information

Supplementary Figures.

## Abbreviations

4HNE: 4-Hydroxynonenal
Aβ: Beta amyloid
AD: Alzheimer’s disease
CA1: Cornus ammonis 1
CA3: Cornus ammonis 3
CNS: Central nervous system
DG: Dentate gyrus
IEM: Immunogold electron microscopy
IMM: Inner mitochondrial membrane
IMS: Intermembrane space
mo: Months-old
OMM: Outer mitochondria membrane
OXPHOS: Oxidative phosphorylation
ROS: Reactive oxygen species
TEM: Transmission electron microscopy
VDAC: Voltage-dependent anionic channel
