# Supplementary material for: Pathologically phosphorylated tau at S396/404 (PHF-1) is accumulated inside of hippocampal synaptic mitochondria of aged Wild-type mice
Source: Sci Rep. 2021 Feb 24;11:4448. doi: 10.1038/s41598-021-83910-w (PMC7904815; doi:10.1038/s41598-021-83910-w)
Supplement: Supplementary file 1 — Supplementary Figures. [file 41598_2021_83910_MOESM1_ESM.pptx]

## Slide 1
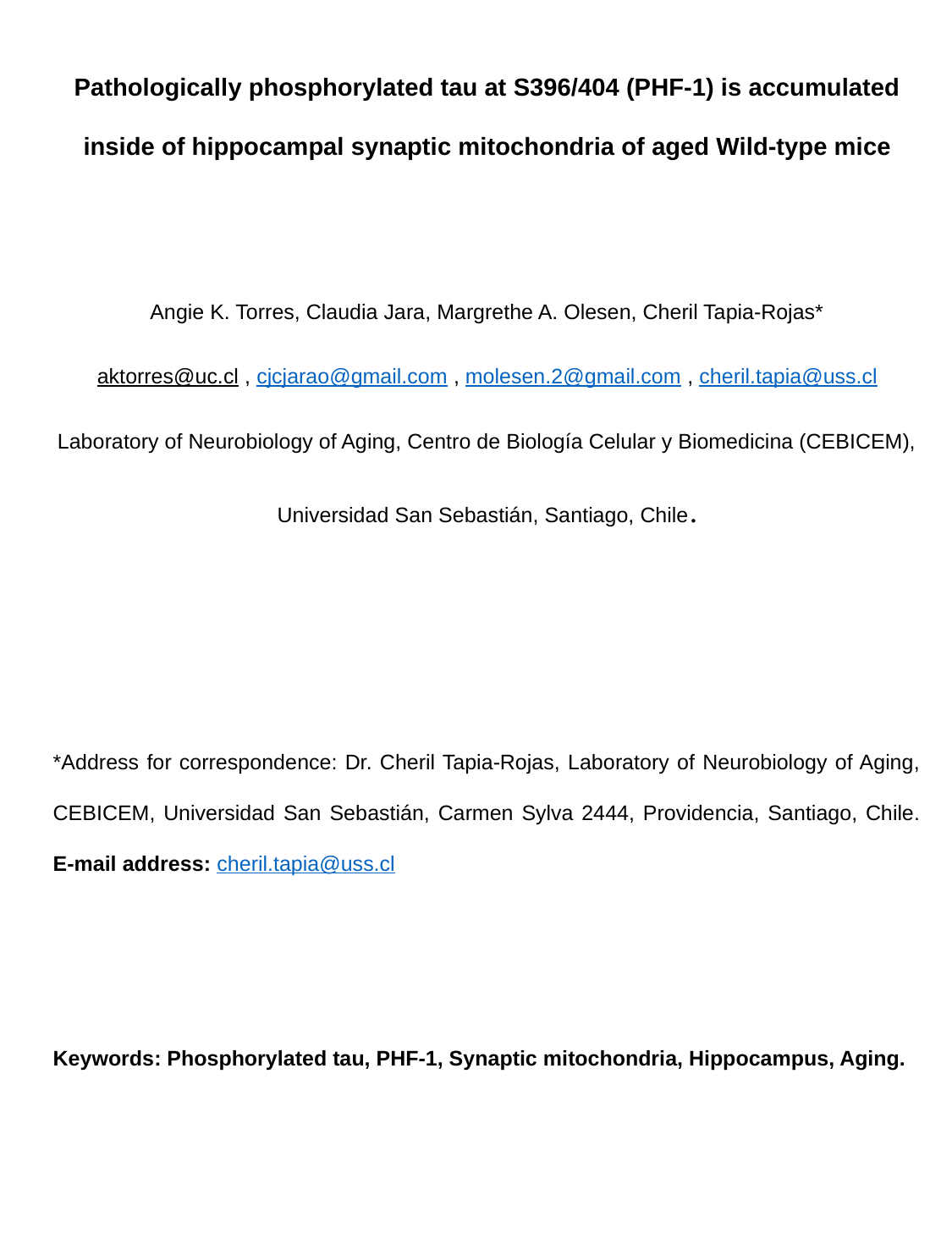

Pathologically phosphorylated tau at S396/404 (PHF-1) is accumulated inside of hippocampal synaptic mitochondria of aged Wild-type mice
Angie K. Torres, Claudia Jara, Margrethe A. Olesen, Cheril Tapia-Rojas*
aktorres@uc.cl , cjcjarao@gmail.com , molesen.2@gmail.com , cheril.tapia@uss.cl
Laboratory of Neurobiology of Aging, Centro de Biología Celular y Biomedicina (CEBICEM), Universidad San Sebastián, Santiago, Chile.
*Address for correspondence: Dr. Cheril Tapia-Rojas, Laboratory of Neurobiology of Aging, CEBICEM, Universidad San Sebastián, Carmen Sylva 2444, Providencia, Santiago, Chile. E-mail address: cheril.tapia@uss.cl
Keywords: Phosphorylated tau, PHF-1, Synaptic mitochondria, Hippocampus, Aging.

## Slide 2
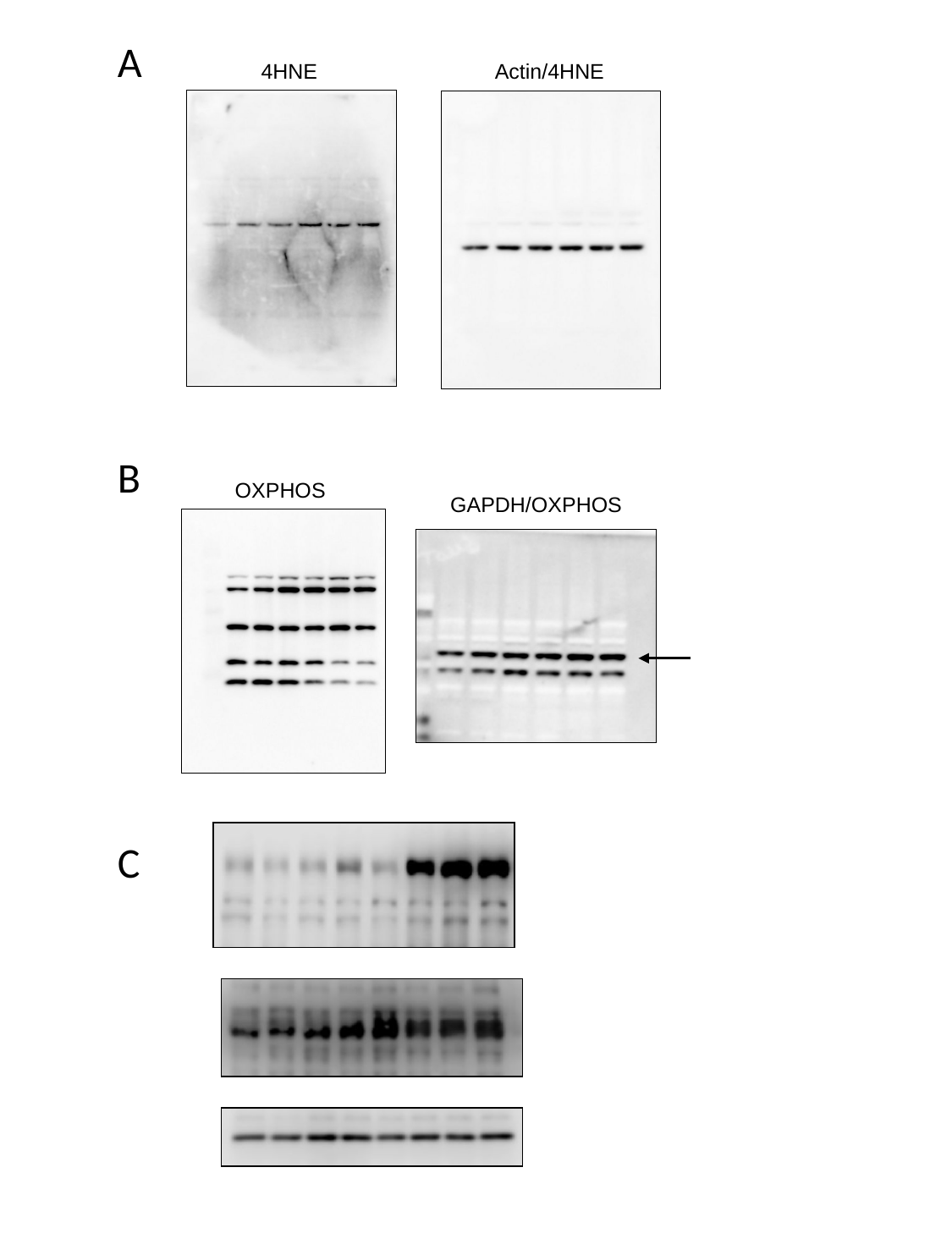

A
Actin/4HNE
4HNE
B
OXPHOS
GAPDH/OXPHOS
C

## Slide 3
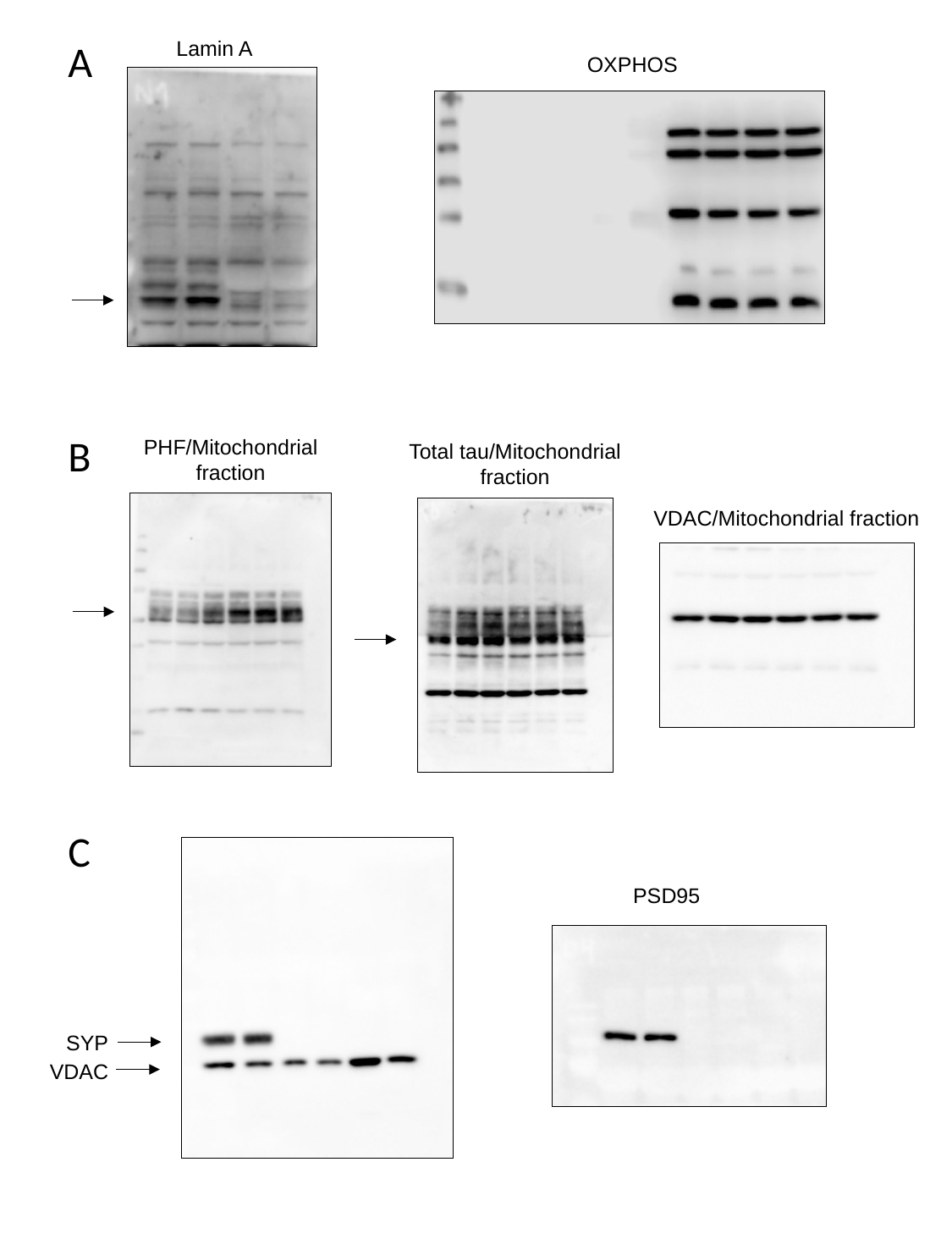

A
Lamin A
OXPHOS
B
PHF/Mitochondrial fraction
Total tau/Mitochondrial fraction
VDAC/Mitochondrial fraction
C
PSD95
SYP
VDAC

## Slide 4
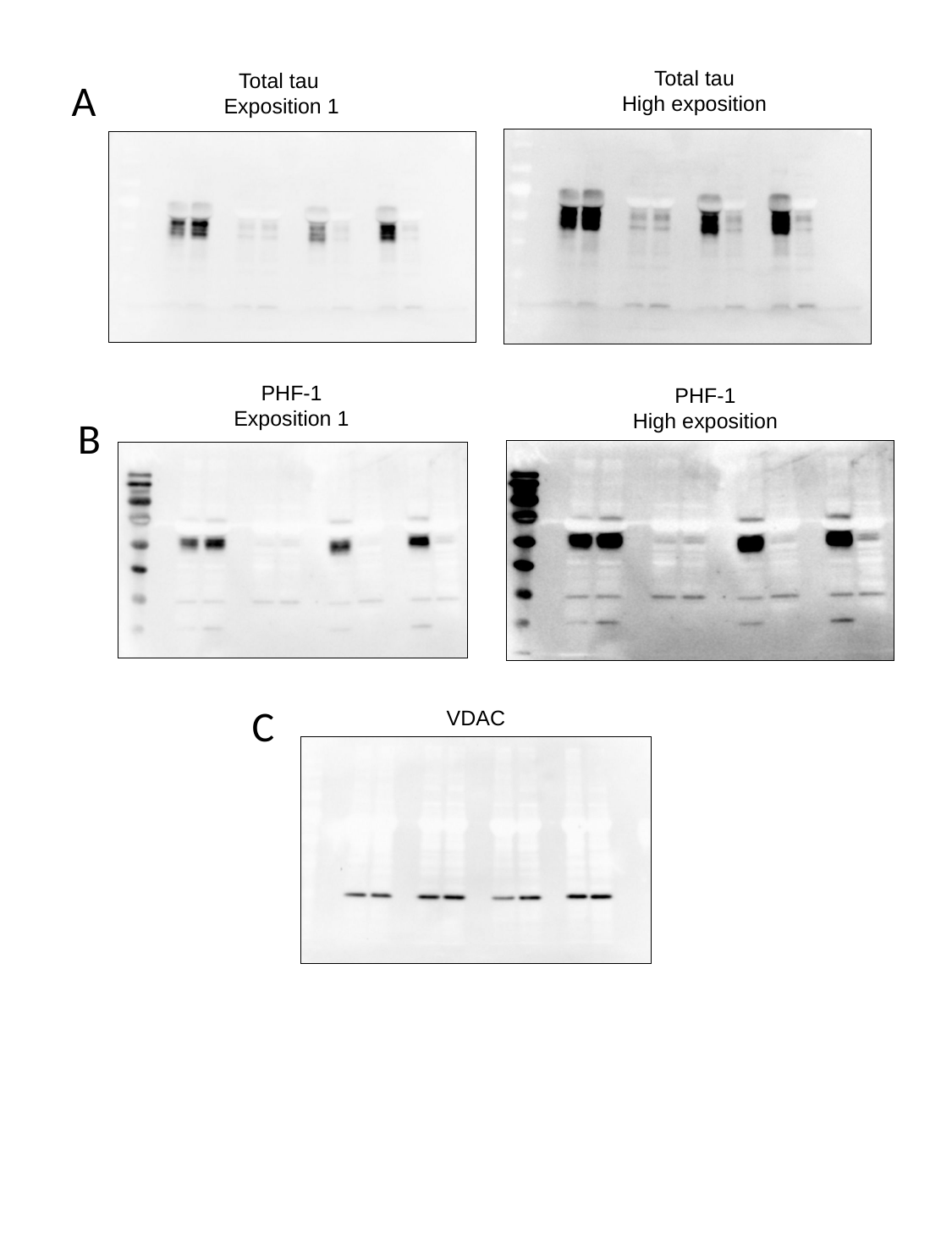

Total tau
High exposition
Total tau
Exposition 1
A
PHF-1
Exposition 1
PHF-1
High exposition
B
C
VDAC

## Slide 5
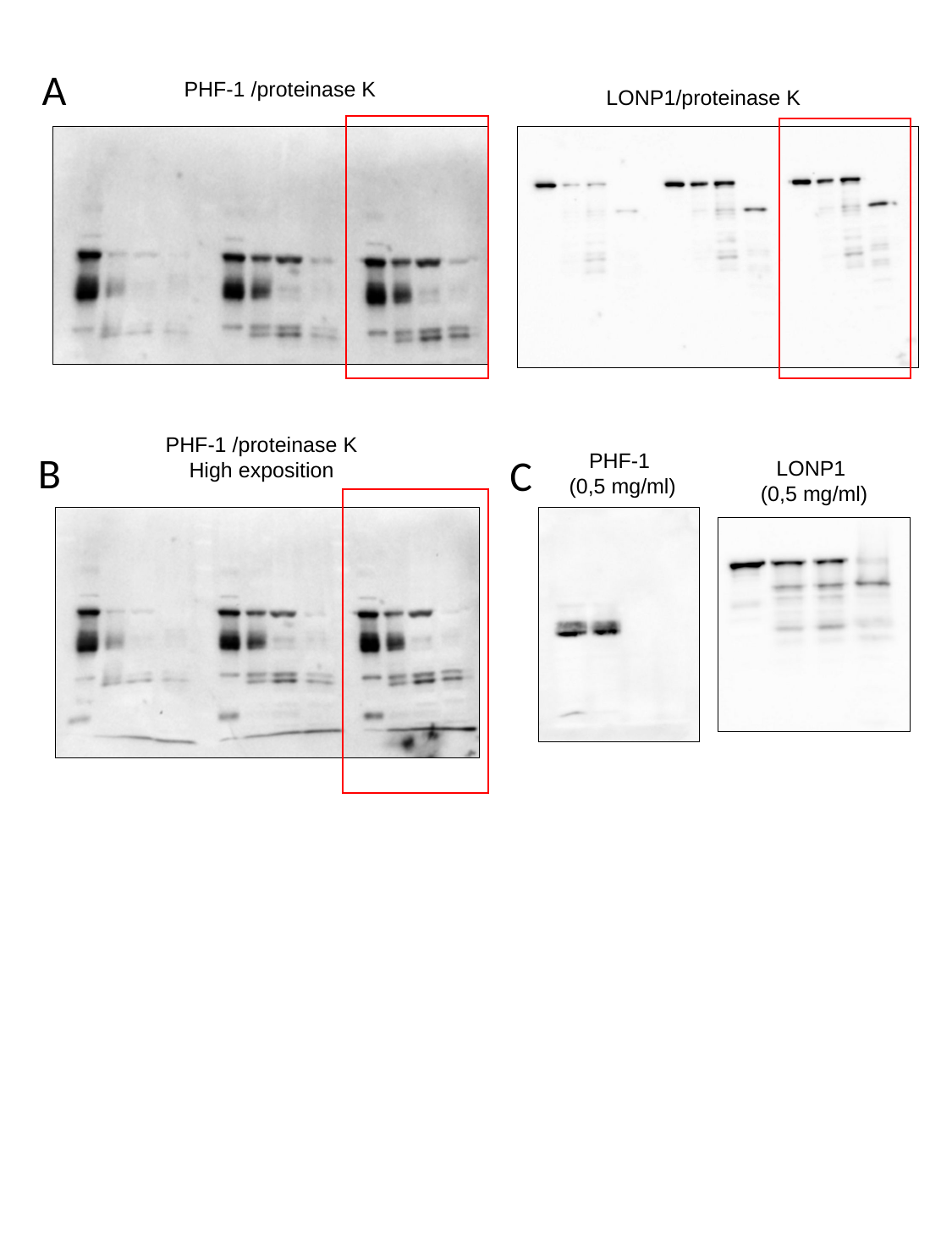

A
PHF-1 /proteinase K
LONP1/proteinase K
PHF-1 /proteinase K
High exposition
B
PHF-1
(0,5 mg/ml)
C
LONP1
(0,5 mg/ml)
